# Distinctive features and prognostic utility of neutrophil in severe patients with *Klebsiella pneumoniae* infection

**DOI:** 10.3389/fcimb.2024.1406168

**Published:** 2024-09-03

**Authors:** Chunjing Du, Ming Lu, Jiajia Zheng, Chao Liu, Ping Yang, Juan Yi, Liuluan Zhu, Ning Shen

**Affiliations:** ^1^ Department of Pulmonary and Critical Care Medicine, Peking University Third Hospital, Beijing, China; ^2^ Center of Infectious Disease, Peking University Third Hospital, Beijing, China; ^3^ Department of Infectious Diseases, Peking University Third Hospital, Beijing, China; ^4^ Department of Laboratory Medicine, Peking University Third Hospital, Beijing, China; ^5^ Institute of Medical Technology, Peking University Health Science Center, Beijing, China; ^6^ Beijing Key laboratory of Emerging Infectious Diseases, Institute of Infectious Diseases, Beijing Ditan Hospital, Capital Medical University, Beijing, China

**Keywords:** K. pneumoniae, neutrophil, infection, innate immunity, prognosis, severe patients

## Abstract

**Background:**

Neutrophil plays a pivotal role in the management of *Klebsiella pneumoniae* infection. Delineate the clinical characteristics and prognostic utility of neutrophil in severe patients with *K. pneumoniae* infection are crucial for clinical management and prognostic assessment.

**Methods:**

*K. pneumoniae* patients with different infection sites were enrolled from Medical Information Mart for Intensive Care IV and eICU Collaborative Research Database. Temporal variations of neutrophil counts within 30 days of clinical onset were examined using locally weighted scatterplot smoothing curves. Logistic regression analysis was performed to assess the relationship between neutrophil counts and hospital mortality.

**Results:**

A total of 1,705 patients caused by *K. pneumonia* were included in the study. The non-survivor group exhibited a comparatively older age and a higher proportion of *K. pneumoniae* infections originating from respiratory and bloodstream sources compared to the survivor group (38.4% vs 21.1%, p<0.0001, and 15.1% vs 10.3%, p=0.021). Patients combined with multiple drug resistance strains, respiratory infection, liver disease, and above 60 years exhibited a specific dynamic process of neutrophil levels. Neutrophils counts peaked at admission and 1-2 weeks later. There was a ‘U’-shaped relationship between neutrophil counts and hospital mortality.

**Conclusions:**

Neutrophils in *K. pneumoniae* infected patients have distinctive features and dynamic clinical trajectories. Close monitoring of severe patients infected with *K. pneumoniae* upon admission and during the first 1-2 weeks after admission is of utmost importance, particularly for patients with a neutrophil count exceeding 8.0×10^9^/L.

## 1 Introduction

*Klebsiella pneumoniae* is a prevalent Gram-negative bacterium known for its ability to cause various infectious diseases, including pneumonia, hepatic abscess, bloodstream infections, and urinary tract infections (Holmes et al., 2021). *K. pneumoniae*, first identified by Carl Friedländer in 1882 as a causative agent of pneumonia, continues to be a prevalent nosocomial pathogen globally (Pendleton et al., 2013). It remains one of the world’s most prevalent nosocomial pathogens and is a leading cause of neonatal sepsis, ranking among the top three causative agents in most settings (Okomo et al., 2019). Notably, *Klebsiella* species are particularly notorious for their ability to develop resistance to multiple antibiotics, including extended-spectrum β-lactam (ESBL) and carbapenems. The World Health Organization recognizes ESBL-producing and carbapenem-resistant *K. pneumoniae* (CRKP) as a critical public health threat (Tacconelli, 2017). In Europe alone, *K. pneumoniae* strains reportedly account for >90,000 infections, >7,000 deaths annually and 25% of the total disability-adjusted life years lost to multidrug-resistant (MDR) bacterial infections, resulting in poorer clinical outcomes (Musicha et al., 2017). The host’s immune response, as a crucial of infectious disease outcomes, is crucial to pathogen clearance and preventing disseminated infection (Metzemaekers et al., 2023; van der Geest et al., 2023). A thorough understanding the clinical characteristics of immune cells, especially during *K. pneumoniae* infections, is crucial for optimizing clinical management and improving patient outcomes.

Neutrophils, the front-line soldiers of the innate immune system, are known for their pivotal role in pathogen elimination (Burn et al., 2021; Tsai et al., 2021; Fine et al., 2020). They are equipped with various bactericidal mechanisms, including phagocytosis, degranulation, and the release of reactive oxygen species and neutrophil extracellular traps (Liew and Kubes, 2019). Neutrophils recognize *K. pneumoniae* through pattern recognition receptors (PRRs) that detect pathogen-associated molecular patterns (PAMPs). This recognition triggers an inflammatory response, leading to the activation of neutrophils and the release of cytokines and chemokines that recruit more immune cells to the site of infection (Chalifour et al., 2004). Increased neutrophil levels frequently serve as a critical indicator of bacterial infection, reflecting the body’s immune response to pathogenic invasion. However, the role of neutrophils during infectious diseases is intricate (Koenderman et al., 2022; Metzemaekers et al., 2020). While neutrophils can effectively eliminate pathogens, they can also create a microenvironment conducive to pathogen survival and proliferation (Metzemaekers et al., 2020; Xu et al., 2021). In addition, sustained elevations in neutrophil counts contribute to tissue damage, exacerbate disease’s severity, and worsen clinical prognosis (Pechous, 2017; Hou et al., 2018; Nguyen et al., 2020). Thus, elucidating the multifaceted roles of neutrophils during infectious diseases are of paramount importance, offering insights that can enhance clinical management and improve patient outcomes.

Clinically, the correlation between neutrophil counts and hospital mortality rates has been well-established in patients with infectious diseases (Salciccioli et al., 2015; Liu et al., 2022). In sepsis and bloodstream infections caused by organisms such as Staphylococcus aureus and Escherichia coli, neutrophil counts and their functional status have been correlated with disease severity and patient outcomes (von Köckritz-Blickwede and Winstel, 2022; Bruns et al., 2011). Similarly, in viral infections, particularly influenza and severe acute respiratory syndrome coronavirus 2 (SARS-CoV-2), neutrophilia and neutrophil activation have been associated with worse outcomes and increased mortality (Jimeno et al., 2021). These studies have highlighted the importance of neutrophils in the pathogenesis of these infections, as well as their potential prognostic value. However, the specific role and prognostic value of neutrophils in infections caused by *K. pneumoniae* have not been as extensively studied. Moreover, there is a lack of data that specifically delineates the clinical profiles of neutrophils during *K. pneumoniae* infections, especially among critically ill individuals whose immune response is frequently dysregulated and may exhibit distinct neutrophil dynamics. Giving the importance of understanding neutrophil clinical attributes for effective clinical management, there is a pressing need for focused studies that explore the behavior of neutrophils and their influence on outcomes in severe patients with *K. pneumoniae* infections.

In the present study, we present a detailed analysis of neutrophil characteristics and clinical trajectory in severe patients with *K. pneumoniae* infections using two extensive databases: the Medical Information Mart for Intensive Care (MIMIC)-IV and the eICU Collaborative Research Database (eICU-CRD). Moreover, we thoroughly investigated the association between neutrophil levels and mortality among the enrolled patients, which holds significant implications for prognostic assessment and clinical management.

## 2 Methods

### 2.1 Data description

In this study, we collected data from two databases of patient cohorts: MIMIC-IV v2.0, and the eICU-CRD v2.0. MIMIC-IV v2.0 (https://physionet.org/content/mimiciv/2.0/) is a publicly available database that consists of 76,540 admissions to the Beth Israel Deaconess Medical Centre (BIDMC; Boston, MA, USA) from 2008 to 2019. The eICU-CRD v2.0 database (https://physionet.org/content/eicu-crd/2.0/) is a multi-center and publicly available dataset that includes over 200,000 discharged patients to ICU across the United States during 2014–2015. Both databases provided demographic details (including age, sex, clinical history, and et al), laboratory measurements, diagnosis, length of stay in the hospital and other comprehensive information. All patient-related information in both databases is anonymous, and no informed consent is required. Data were extracted by author CD (certification number 50491649).

### 2.2 Study population

All patients in the MIMIC-IV v2.0 and eICU-CRD v2.0 databases were eligible for inclusion for the present investigation. The inclusion criteria were as follows: (i) age 18≥ years; (ii) a positive microbial culture for *K. pneumoniae*, obtained within the 48h-period preceding admission; and (iii) a definitive diagnosis of infection, corroborated by microbial culture results and corresponding International Classification of Diseases (ICD)-9 and ICD-10 diagnostic codes. The exclusion criteria included: (i) patients with duplicated records of infection; and (ii) multiple microbiological cultures from the same site; and (iii) incomplete data (individuals having more than 20% of the required variables missing) or absence of neutrophil results (shown as **Supplementary Figure S1**). To manage missing data, we employed the method of multiple imputation. The primary outcome was the in-hospital mortality, while secondary outcomes included ICU mortality, and hospital- and ICU length of stay (LOS).

### 2.3 Data extraction

Patient demographics, including age, gender, ICU and hospital LOS, ICU and hospital mortality rates were extracted as part of the demographic data. Comorbidities such as hypertension, diabetes, chronic pulmonary disease, coronary heart disease, liver disease, renal disease, and malignancy were also recorded. The Charlson index, a widely accepted measure of comorbidity burden, was calculated for each patient. The diagnosis of comorbidities was determined by the recorded ICD-9 and ICD-10 codes. Microbiology and laboratory played a crucial role in our analysis. Detailed information regarding the bacteria isolated, site of infection, and hematological parameters (including white blood cell counts, neutrophil counts, lymphocyte counts, red blood cell counts, and platelet counts) during their hospital stay were collected. In cases where patients had multiple hospitalizations, only the data related to the first hospitalization admission were collected. All enrolled patients had no record of being readmitted to the hospital within 7 days of their discharge due to infection. The data were extracted using Navicate Premium software (version 15.0) through a running Structured Query Language (SQL).

### 2.4 Statistical analysis

Continuous variables were presented as median with interquartile range (IQR) or mean with standard deviation (SD) and their statistical significance was assessed using the *t*-test or Wilcoxon rank sum test. Categorical variables were reported as frequencies and percentages, and their statistical significance was analyzed using the chi-square (χ2) or Fisher’s exact tests. To investigate the relationship between neutrophil counts and hospital mortality rates, as well as the dynamic fluctuations of neutrophils in *K. pneumoniae* infection within 30 days of clinical onset, locally weighted scatterplot smoothing (LOWESS) curves were constructed. Logistic regression models were employed to analyze the impact of neutrophil levels on hospital mortality, using the neutrophil range of 4.8-8.0×10^9^/L serving as the reference group. The stepwise backward elimination method was employed to establish the final logistic regression model, with a significance level of 0.05. All statistical analyses were performed using the Stata (v 17.0) or R (v 4.2.2) software. Two-sided *p*-values <0.05 were considered statistically significant.

## 3 Results

### 3.1 Population and baseline characteristics

A total of 126,420 positive microbiological culture records of *K. pneumoniae* documented in both MIMIC-IV v2.0 database (n=125,440) and the eICU-CRD v2.0 database (n=980). Patient inclusion was grounded in the confirmation of these positive culture results, combined with corresponding diagnostic codes, which collectively indicated a definitive *K. pneumoniae* infection. The final analysis involved 1,705 patients, including 1,434 survivors and 271 non-survivors, with a mortality rate of 15.9% (**Supplementary Figure S1**). In the combined cohort of the two databases, the non-survivor group exhibited a comparatively older age and a higher proportion of *K. pneumoniae* infections originating from respiratory and bloodstream sources compared to the survivor group (38.4% vs 21.1%, *p*<0.0001, and 15.1% vs 10.3%, *p*=0.021, **Table 1**). There were no significant differences in different types of *K. pneumoniae* strains between the survivor group and the non-survivor group (*p*>0.05). In terms of co-morbidities, the non-survival group had a higher incidence of liver disease and kidney disease, along with Charlson index, compared to the survival group. Conversely, the incidence of hypertension in the non-survival group was lower than that observed in the survival group. Additionally, the non-survivor group demonstrated lower urinary infection rates than the non-survivor group (37.3% vs 53.3%, *p*<0.0001), as well as decreased counts of lymphocyte, platelet, RBC, and hemoglobin (**Table 1**). The baseline characteristics of each database are presented in **Supplementary Tables S1**, **S2**.

**Table 1:** Comparisons of baseline characteristics between survivors and non-survivors.

Variable	Total	Survivors	Non-survivors	*P* value
**No. of patients, n (%)**	1705	1434 (84.1)	271 (15.9)	
**Age (years)**	68 (57,78)	68 (56,78)	70 (60,80)	0.004
**Male, n (%)**	827 (48.5)	681 (47.5)	146 (53.9)	0.054
**Ethnicities, n**	1705	1434	271	
Asian, n (%)	62 (3.6)	55 (3.8)	7 (2.6)	0.312
Caucasian/White, n (%)	1149 (67.4)	980 (68.3)	169 (62.4)	0.054
Hispanic, n (%)	69 (4.1)	60 (4.2)	9 (3.3)	0.508
American Indian/Alask, n (%)	6 (0.4)	5 (0.4)	1 (0.4)	0.647
Portuguese, n (%)	7 (0.4)	5 (0.3)	2 (0.7)	0.308
African, n (%)	198 (11.6)	172 (12.0)	26 (9.6)	0.258
Unknown, n (%)	214 (12.6)	157 (10.9)	57 (21.0)	<0.0001
***K. pneumoniae* strains, n**	1117	878	239	
CRKP, n (%)	166 (14.9)	136 (15.5)	30 (12.6)	0.258
ESBL, n (%)	292 (26.1)	223 (25.4)	69 (28.9)	0.279
MDR, n (%)	24 (2.2)	17 (1.9)	7 (2.9)	0.348
Infection site, n (%)				
Respiratory infection	406 (23.8)	302 (21.1)	104 (38.4)	<0.0001
Urinary infection	765 (50.8)	765 (53.3)	101 (37.3)	<0.0001
Bloodstream infection	189 (11.1)	148 (10.3)	41 (15.1)	0.021
Peritoneal/Pleural/Abscess/Bile infection	96 (5.6)	85 (5.9)	11 (4.1)	0.221
Other	148 (8.7)	134 (9.3)	14 (5.2)	0.025
Co-morbidities, n (%)				
Hypertension	633 (37.1)	547 (38.1)	86 (31.7)	0.045
Diabetes	640 (37.5)	537 (37.4)	103 (38.0)	0.861
Chronic pulmonary disease	418 (24.5)	354 (24.7)	64 (23.6)	0.707
Coronary heart disease	534 (31.3)	444 (31.0)	90 (33.2)	0.464
Liver disease	268 (15.7)	197 (13.7)	71 (26.2)	<0.0001
Renal disease	467 (27.4)	378 (26.4)	89 (32.8)	0.028
Malignancy	486 (28.5)	405 (28.2)	81 (29.9)	0.582
**Charlson index**	6.0 (4.0,8.0)	6.0 (4.0,8.0)	7.0 (5.0,9.0)	<0.0001
Laboratory tests				
WBC (×10^9^/L)	9.6 (6.6,13.8)	9.4 (6.5,13.5)	10.5 (7.0,14.5)	0.017
Neutrophil (×10^9^/L)	8.0 (4.8,12.4)	7.8 (4.8,12.0)	9.6 (5.2,14.6)	<0.0001
Lymphocyte (×10^9^/L)	1.1 (0.6,1.7)	1.1 (0.7,1.7)	0.9 (0.5,1.6)	0.001
Platelet (×10^9^/L)	206.5 (136.0,288.0)	211.0 (141.0,290.0)	182.5 (114.0,269.5)	0.002
RBC (×10^12^/L)	3.5 (3.0,4.0)	3.5 (3.0,4.0)	3.3 (2.8,3.8)	<0.0001
Hemoglobin (g/dl)	10.2 (8.8,11.8)	10.3 (8.9,11.9)	9.8 (8.4,11.4)	0.002

CRKP, Carbapenem-Resistant Klebsiella pneumoniae; ESBL, Extended-spectrum Beta-lactamase Resistant Klebsiella pneumoniae; MDR, Multiple Drug Resistance; RBC, red blood cell; WBC, white blood cell.

### 3.2 Differences and dynamic clinical trajectory of neutrophils in patients with *K. pneumoniae* infection among different groups

**Figure 1:**
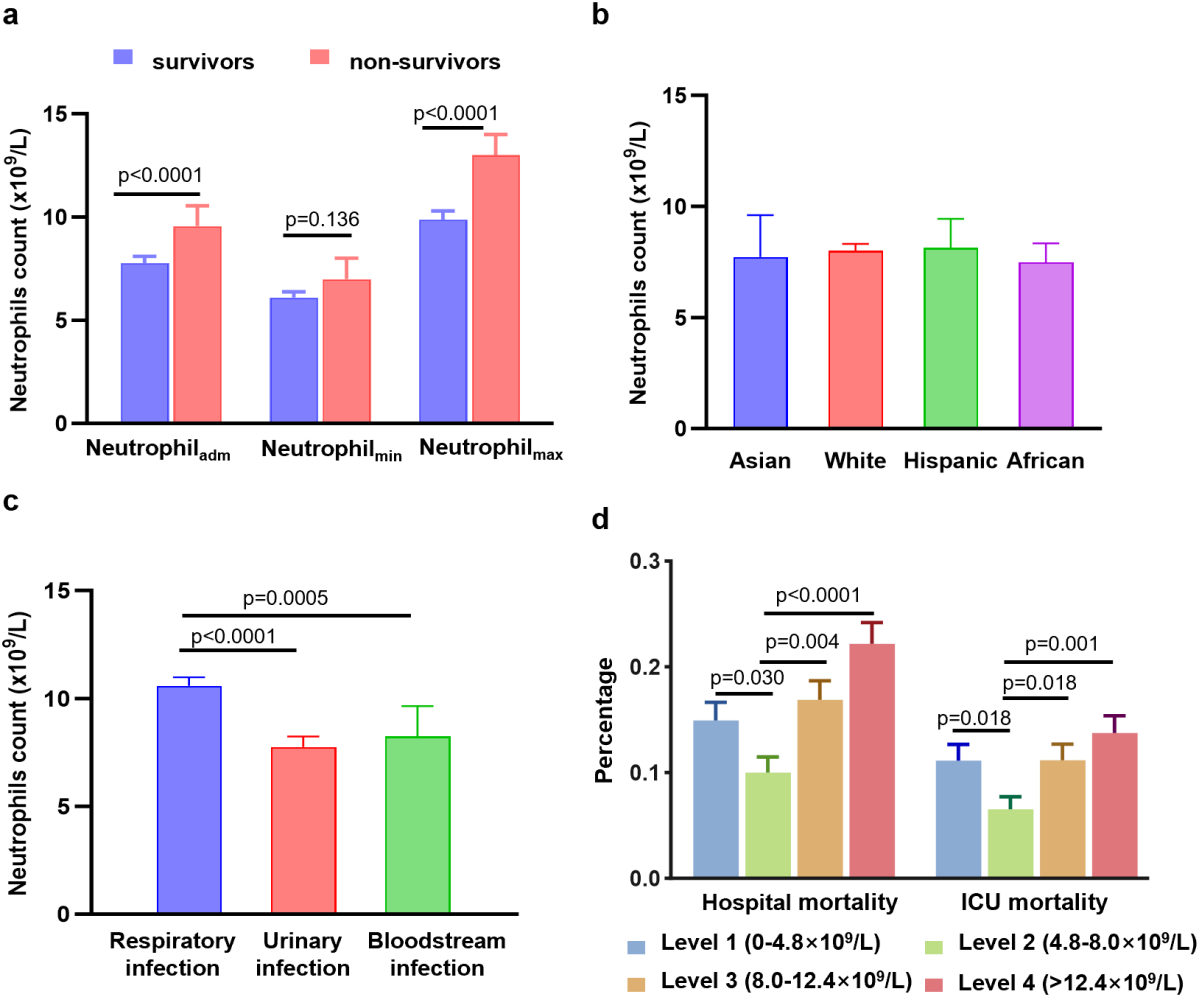
Differences in neutrophil counts, hospital mortality, and ICU mortality among different groups. **(A)** Differences in neutrophil counts at the admission values, minimum values, and maximum values between the survivor group and non-survivor group. **(B)** Differences in neutrophil counts among different ethnicities. **(C)** Differences in neutrophil counts among different infection sites. **(D)** Differences in hospital mortality and ICU mortality among four level groups of neutrophils. Data are presented as media (95% CI).

**Figure 2:**
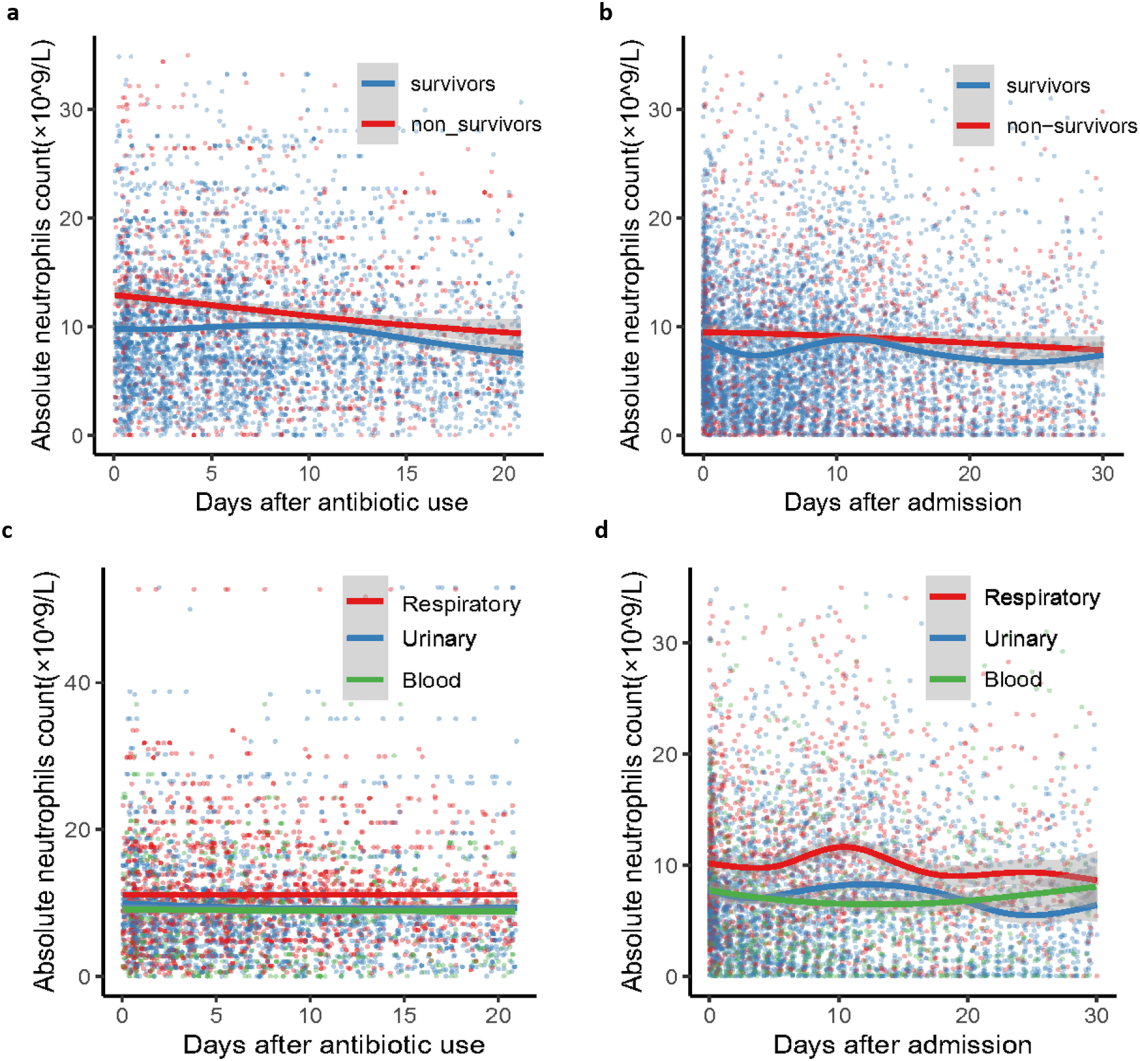
Dynamic clinical trajectory of neutrophil counts among survivor group, non-survivor group and groups of *K*. *pneumoniae* infected patients with respiratory, blood and urinary. **(A)** survivor group and non-survivor group of neutrophil dynamic clinical trajectory after antibiotic use. **(B)** survivor group and non-survivor group of neutrophil dynamic clinical trajectory after admission. **(C)** *K*. *pneumoniae* infected patients with respiratory, blood and urinary groups of neutrophil dynamic clinical trajectory after antibiotic use. **(D)** D *K*. *pneumoniae* infected patients with respiratory, blood and urinary groups of neutrophil dynamic clinical trajectory after admission. The Confidence interval (CI) at 95% is indicated as a gray shadow.

**Figure 3:**
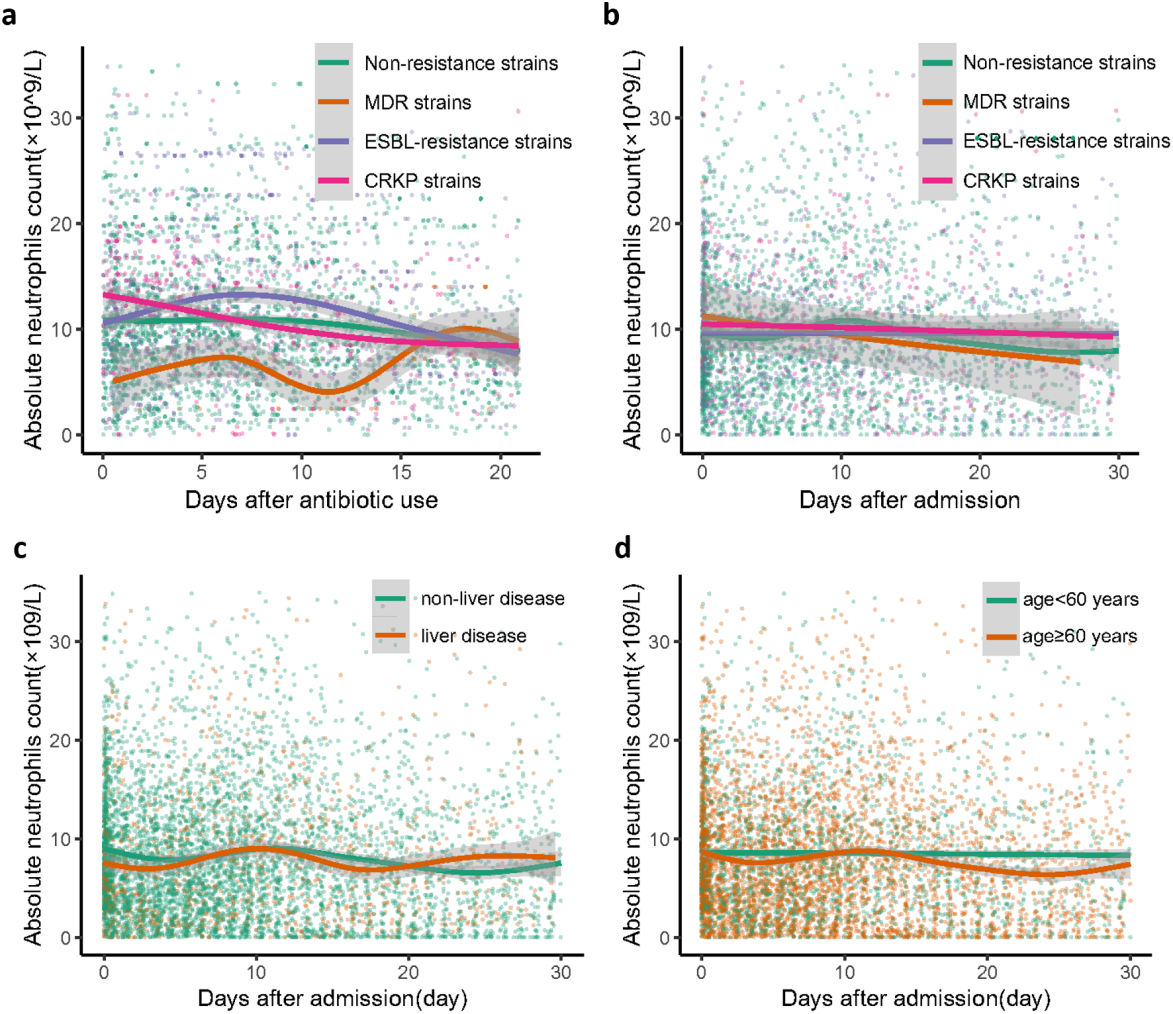
Dynamic clinical trajectory of neutrophil counts among different *K*. *pneumoniae* strains and different combination situations. **(A)** different *K*. *pneumoniae* strains of neutrophil dynamic clinical trajectory after antibiotic use. **(B)** different *K*. *pneumoniae* strains of neutrophil dynamic clinical trajectory after admission. **(C)** non-liver disease group and liver disease group of neutrophil dynamic clinical trajectory after admission. **(D)** age <60 years group and age ≥60 years group of neutrophil dynamic clinical trajectory after admission. The Confidence interval (CI) at 95% is indicated as a gray shadow. CRKP, Carbapenem-Resistant Klebsiella pneumoniae; ESBL, extended-spectrum beta-lactamase; MDR, multiple drug resistance.

Neutrophil counts from the enrolled patients throughout their 30-day hospitalization were analyzed to investigate the characteristics and dynamic profiles among groups in different infection sites and survival conditions. The non-survivor group exhibited significantly higher neutrophil values at admission (*p*<0.0001), as well as at minimum and maximum levels (*p*=0.136 and *p*<0.0001, respectively), compared to the survivor group (**Figure 1A**). As shown in **Figures 2A, B**; **Supplementary Figures S2A, B**, the non-survivor group displayed a relatively high levels of neutrophil levels compared the survivor group no matter after antibiotics use or admission. In addition, the survivor group demonstrated a fluctuating pattern with a peak occurring approximately two weeks after admission. There were no significant differences in neutrophil values among different ethnicities (**Figure 1B**). In terms of different infection sites, the respiratory infection group had significantly higher neutrophil values compared to the urinary infection group and bloodstream infection group (*p*<0.0001 and *p*=0.0055, respectively, **Figure 1C**). Following antibiotic administration, the disparity in neutrophil levels among the three groups diminished (**Figures 2C, D**). Furthermore, patients combined with multiple drug resistance (MDR) strains, liver disease, above 60 years, and respiratory infection exhibited a specific dynamic process of neutrophil levels. In detail, their neutrophil counts increased within the first week, gradually declining after reaching a peak at approximately 10 days following admission (**Figures 3A–D**; **Supplementary Figures S2C-D**, **S3**, **S4A-C**). These dynamic profiles provide important insights into the temporal changes in neutrophil counts and their association with patient outcomes.

### 3.3 Association between neutrophil counts and hospital mortality in patients with *K. pneumoniae* infection

**Figure 4:**
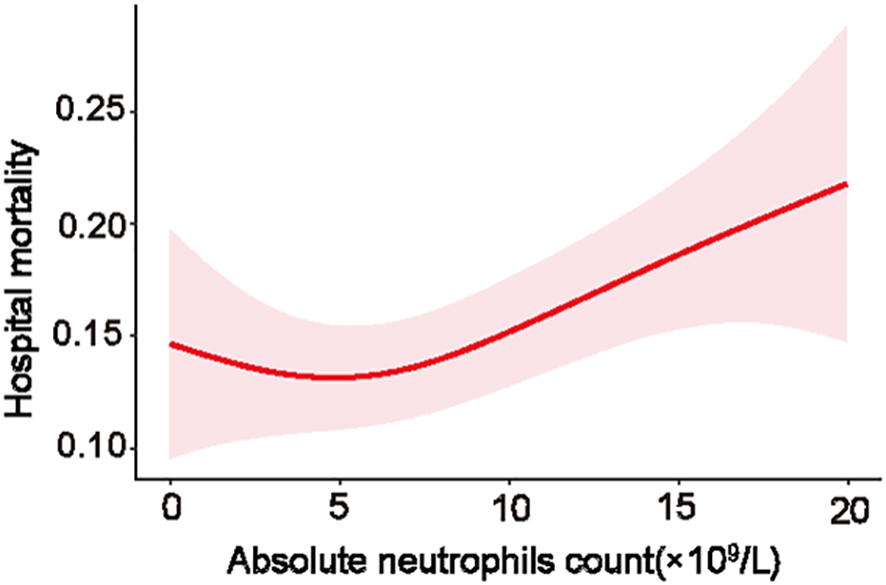
Statistical probability of hospital mortality vs neutrophils count.

To further explore the relationship between neutrophil count and hospital mortality in patients infected with *K. pneumoniae*, a LOWESS curve analysis was conducted (**Figure 4**). Results revealed a ‘U’-shaped relationship, indicating that hospital mortality rates were lowest when the neutrophil count was 5.0×10^9^/L. Subsequently, we divided the neutrophil levels into four groups based on the median and IQR, with the reference group set as 4.8-8.0×10^9^/L for all comparisons and logistic regression models. Despite not statistically significant, the hospital mortality and ICU mortality rates were higher in level 1, level 3, and level 4 groups than the reference group of level 2 (**Figure 1D**; **Supplementary Figure S4D**). Univariate analysis revealed that higher neutrophil levels were associated with an increased risk of hospital mortality, with the OR increasing stepwise from level 1 (OR: 1.58; [95% CI 1.04 - 2.38], *p*=0.031) to level 4 (OR: 2.5; [95% CI 1.69 - 3.68], *p*<0.0001). These trends were consistently observed in each individual database (**Table 2**; **Supplementary Table S3**).

**Table 2:** Univariate logistic regression analysis using neutrophil for hospital mortality.

Variables (different groups of neutrophils, ×10^9^/L)	Crude odds ratio	95% CI	P value
Level 1 (0-4.8)	1.58	1.04-2.38	0.031
Level 2 (4.8-8.0)	Ref.		
Level 3 (8.0-12.4)	1.82	1.21-2.72	0.004
Level 4 (≥12.4)	2.50	1.69-3.68	<0.0001

### 3.4 Multivariate logistic analysis for the risk factors of hospital mortality in patients with *K. pneumoniae* infection

To determine whether the neutrophil count independently influenced hospital mortality in patients infected with *K. pneumoniae*, a logistic regression model was employed. Each group of neutrophils levels was analyzed after adjusting for covariates with *p* values of <0.05 in univariate analyses, including age, Charlson’s comorbidity index, comorbidities, and different routes of infection. Using level 2 used as the reference group, the logistic regression analysis revealed that the neutrophil levels were an increased risk of hospital mortality. The odds ratio (OR) showed a stepwise increment from level 3 (OR: 1.53; 95% CI 1.09-2.16) to level 4 (OR: 2.01; 95% CI 1.45-2.79) (**Table 3**). These results were consistent with the findings obtained from the MIMIC database (**Supplementary Table S3**). Notably, a neutrophil count of ≥8.0×10^9^/L was identified as an independent risk factor for hospital mortality in patients with *K. pneumoniae* infection. This suggests that higher neutrophil levels are associated with an increased likelihood of hospital mortality in this patient population.

**Table 3:** Adjusted odds ratio using neutrophil as the design variable in multivariate logistic regression.

Variable	Odds ratio	95% CI	P value
Level 3 (8.0-12.4×10^9^/L)	1.53	1.09-2.16	0.015
Level 4 (≥12.4×10^9^/L)	2.01	1.45-2.79	<0.0001
Respiratory infection	2.70	1.99-3.67	<0.0001
Bloodstream infection	2.00	1.33-3.02	0.001
RBC	0.74	0.61-0.90	0.002
Age	1.01	1.00-1.02	0.038
Liver disease	1.96	1.37-2.80	<0.0001
Charlson comorbidity index	1.12	1.06-1.19	<0.0001

RBC, red blood cell.

## 4 Discussion

*K. pneumoniae* infections pose a significant challenge due to its elevated morbidity and mortality rates. Considering the limited clinical understanding of neutrophils’ involvement in such infections, we aimed to address this knowledge gap by comprehensive analyses using two large clinical databases. Our findings revealed that non-survivors had significantly higher neutrophil counts compared to survivors. Patients with respiratory infections, without liver disease, and below 60 years of age exhibited higher neutrophil levels and had different dynamic profiles. The peak levels of neutrophils were observed at the time of admission or approximately 1-2 weeks later. Importantly, neutrophil counts appeared to follow a “U”-shaped relationship between neutrophil counts and hospital mortality. Patients with a neutrophil count ≥ 8.0×10^9^/L emerged as a subgroup of particular concern during hospitalization, as this parameter was identified as an independent risk factor for hospital mortality in patients with *K. pneumoniae* infection. These findings contribute to our understanding of the pathophysiology of *K. pneumoniae* infections and assist clinicians in identifying patients who may require closer monitoring and more intensive management strategies.

Our findings revealed a crucial aspect regarding the timing of neutrophil peaks, which occurred at the time of admission or approximately 1-2 weeks later. This observation reflects a dynamic pattern of neutrophil response during the course of infection and holds significant clinical implications in the management of *K. pneumoniae* infected patients. In the human body, the circulatory lifespan of neutrophils averages around 5.4 days (Pillay et al., 2010). However, during inflammatory processes, the longevity of neutrophils can be magnified manifold, ensuring the recruitment of primed neutrophils to the precise sites of inflammation (Koenderman et al., 2022). Elevated levels of neutrophils remained detectable even at 7 to 14 days post-infection. Of particular interest, the rapid recruitment of neutrophils within the initial 48 hours following infection does not exhibit a direct correlation with a reduction in bacterial burden (Ahn et al., 2016; Peñaloza et al., 2019; Wang et al., 2020). The resolution of inflammation involves a multifaceted interplay between anti-inflammatory and pro-resolving phases, which can influence the generation and recruitment of neutrophils. This intricate balance between pro-inflammatory and anti-inflammatory factors throughout the progression of infection, coupled with the re-emergence of pathogens, likely contributes to the dynamic profiles exhibited by neutrophils (Ji and Fan, 2021; Bruserud et al., 2023).

The observation of a distinctive ‘U’-shaped relationship between neutrophil levels and hospital mortality is an interesting finding, which suggests that there might be an optimal range of neutrophil counts for fighting *K. pneumoniae* infections. Notably, while higher and lower levels of neutrophils were both correlated with increased mortality rates, this association does not necessarily imply a direct causative relationship. In present study, non-survivors exhibited higher neutrophil counts than survivors. Furthermore, patients with elevated or decreased neutrophil levels (groups of level 1, 3, and 4) exhibited increased hospital and ICU mortalities compared to the reference group (group of level 2). This finding likely reflects the intricate interplay between neutrophils and other factors in the context of severe infections. While an increase in neutrophil counts, or neutrophilia, is a typical response to bacterial infections like *K. pneumoniae* and is generally perceived as advantageous as it indicates the host’s active immune response, the scenario in severe infections is less clear-cut. In such cases, very severe infections might precipitate either an increase or a decrease in neutrophil counts and functions. This can lead to an immune system malfunction, coupled with multiple organ dysfunction, which may further intensify disease progression (Pechous, 2017; Metzemaekers et al., 2020; Zanza et al., 2022). As neutrophil counts are influenced by a multitude of factors, including the severity of the infection, the host’s immune response, and the overall clinical condition, it is essential to interpret neutrophil levels within the broader clinical context.

Our study identified neutrophil counts were independent risk factors for hospital mortality. This finding aligns with previous research (Lv et al., 2022). Importantly, we validated a critical cut-off value at 8.0×10^9^/L, which serves as significant biomarker for differentiating patients at increased risk of mortality due to elevated neutrophil levels. These findings emphasize the necessity for close surveillance of neutrophil dynamics in the clinical setting, especially in cases where they exceed the established cut-off. Integrating this biomarker with other clinical data empower healthcare professionals to make more informed decisions about patient management. This facilitates the implementation of targeted therapeutic interventions, encompassing early antimicrobial therapy and adjunctive treatments, thereby optimizing patient care and outcomes.

In current study, patients with respiratory infections caused by *K. pneumonia* exhibited higher levels of neutrophils compare to those with bloodstream infections and other sites of infections. Neutrophils in *K. pneumoniae* patients with different infection sites have distinctive features and dynamic clinical trajectories. Previous studies have demonstrated that the lungs serve as a natural reservoir of mature neutrophils, which can be rapidly mobilized to sites of inflammation or infection (Ostrand-Rosenberg et al., 2023). Furthermore, it has been suggested that all blood neutrophils undergo a temporary pulmonary sequestration due to the lungs receiving the entire cardiac output (Summers et al., 2010; Fay et al., 2016; Pararajasingam et al., 1999). Considering these factors, the higher neutrophil levels observed in patients with respiratory infections may be attributed to the recruitment and accumulation of neutrophils in the lung tissues. Since neutrophil counts have been identified as an independent risk factor for hospital mortality, it is necessary to promptly identify the site of infection, particularly focusing on patients with respiratory infections.

In present study, patients who had liver disease or were aged ≥ 60 years displayed lower neutrophil levels in comparison to the control group consisting of patients without liver disease or younger than 60 years old. Concurrently, we conducted a comprehensive analysis, comparing and illustrating the fluctuations of neutrophil levels across both groups. This analysis provided a detailed depiction of neutrophil dynamics in relation to liver disease status and age demographics. The liver plays a central role as an immunological organ, housing various myeloid immune cells, including neutrophils, macrophages, and Kupffer cells (Kubes and Jenne, 2018; Ahmed et al., 2021). Therefore, the presence of liver disease may contribute to a decrease in neutrophil levels. It is important to note that while the absolute number of neutrophils does not vary significantly with age, there is evidence to suggest that aging is associated with impaired phagocytosis, chemotaxis, and superoxide generation functions of neutrophils (Hornigold et al., 2022; Lewis et al., 2022). Additionally, elderly patients may experience decreased anti-pathogen activity due to increased apoptosis of neutrophils, resulting in a higher susceptibility to more frequent and severe infections (Hornigold et al., 2022; Wen et al., 2022).

Despite significant findings regarding the dynamic profiles of neutrophils and their association with hospital mortality, several potential limitations in this study should be acknowledged. Firstly, retrospective analyses inherently possess limitations in establishing causal associations definitively. A more comprehensive understanding of the immunological mechanisms encompassing both the quantitative dynamics and functional integrity of neutrophils is warranted. Secondly, the database lacked comprehensive data on inflammatory biomarkers such as C-reactive protein and procalcitonin, which precluded any in-depth analysis or comparison of these markers with neutrophil counts in this study. Thirdly, while our findings are focused on primary *K. pneumoniae* infections, the potential influence of coinfections and reinfections on neutrophil counts was not addressed. Further studies should take these factors into account to refine the understanding of neutrophil dynamics in the context of infectious diseases. Lastly, while the statistical association between hospital mortality and neutrophil levels has been demonstrated, it is essential to include data from other clinical databases that help confirm the robustness of the associations. Meanwhile, its practical application in clinical practice should be evaluated across different clinical scenarios.

In summary, neutrophils in *K. pneumoniae* infected patients have distinctive features and dynamic clinical trajectories. Closely monitoring severe patients infected with *K. pneumoniae* upon admission and during the first 1-2 weeks after admission is of utmost importance. This is particularly significant for patients with a neutrophil count exceeding 8.0×10^9^/L, as elevated neutrophils levels were correlated with a poor prognosis in individuals with *K. pneumonia* infection. To effectively manage severe cases of *K. pneumoniae* infections, it is essential to further investigate the intricate mechanisms of neutrophils during *K. pneumoniae* infections.

## Data Availability

Publicly available datasets were analyzed in this study. This data can be found here: https://physionet.org/content/mimiciv/2.0/ https://physionet.org/content/eicu-crd/2.0/.
