# Supplementary material for: Distinctive features and prognostic utility of neutrophil in severe patients with Klebsiella pneumoniae infection
Source: Front Cell Infect Microbiol. 2024 Sep 3;14:1406168. doi: 10.3389/fcimb.2024.1406168 (PMC11405363; doi:10.3389/fcimb.2024.1406168)
Supplement: Supplementary file 1 [file DataSheet1.docx]

**Distinctive features and prognostic utility of neutrophil in severe patients with Klebsiella pneumoniae infection**

**Supplementary files**

**Supplementary File 1: Table S1.** Baseline characteristics of each database.

**Supplementary File 2: Table S2.** Comparisons of baseline characteristics between survivors and non-survivors in each database.

**Supplementary File 3: Table S3.** Univariate logistic regression analysis using neutrophil for hospital mortality in each database.

**Supplementary File 4: Table S4.** Adjusted odds ratio using neutrophil as the design variable in multivariate logistic regression of Mimic database.

**Supplementary File 5: Fig. S1** Flow chart of steps used for patient selection.

**Supplementary File 6: Fig. S2** Dynamic clinical trajectory of neutrophil counts among survivor group, non-survivor group, and different infectious pathways in each database. Neutrophil counts in the survivor group vs non-survivor group in MIMIC database (**a**) and eICU-CRD database (**b)**. Neutrophil counts among in *K. pneumoniae* infected patients with respiratory, blood, urinary, peritoneal/pleural/ abscess/bile symptoms, vs other pathways in MIMIC database (**c**) and eICU-CRD database (**d)**. CI at 95% is indicated as a gray shadow.

**Supplementary File 6: Fig. S3** Dynamic clinical trajectory of neutrophil counts among non-liver disease, liver disease and different age groups in each database. Neutrophil counts in non-liver disease group vs liver disease group in MIMIC database (**a**) and eICU-CRD database (**b)**. Neutrophil counts in age< 60 years vs age≥60 years in MIMIC database (**c**) and eICU-CRD database (**d)**. CI at 95% is indicated as a gray shadow.

**Supplementary File 7: Fig. S4** Dynamic clinical trajectory of neutrophil counts among different *K. pneumoniae* strains and differences of different neutrophil levels in the hospital LOS and ICU LOS. (**a)** non-MDR strains and MDR strains of neutrophil dynamic clinical trajectory after antibiotic use. (**b)** non-CRKP strains and CRKP strains of neutrophil dynamic clinical trajectory after antibiotic use. (**c)** non-ESBL resistance strains and ESBL resistance strains of neutrophil dynamic clinical trajectory after antibiotic use. The Confidence interval (CI) at 95% is indicated as a gray shadow. **(d)** Differences in the hospital LOS and ICU LOS in relation to different levels of neutrophils. Data are presented as media (95% CI). CRKP, Carbapenem-Resistant Klebsiella pneumoniae; ESBL, extended-spectrum beta-lactamase; LOS, length of stays; MDR, multiple drug resistance.

Table S1. Baseline characteristics of each database.

| Variable | Mimic | eICU |
| --- | --- | --- |
| **No. of patients, n (%)** | 1633(100) | 72(100) |
| **Age (years)** | 68(56,78) | 69(58,75) |
| **Male, n (%)** | 794(48.6) | 33(45.8) |
| **Infection site, n (%)** |  |  |
| Respiratory infection | 376(23.0) | 30(41.7) |
| Urinary infection | 835(51.1) | 31(43.1) |
| Blood infection | 184(11.3) | 5(6.9) |
| Peritoneal/Pleural/Abscess/Bile infection | 94(5.8) | 2(2.8) |
| Other | 144(8.8) | 4(5.6) |
| **Comorbidities, n (%)** |  |  |
| Hypertension | 616(37.7) | 17(23.6) |
| Diabetes | 618(37.8) | 22(30.6) |
| Chronic pulmonary disease | 403(24.7) | 15(20.8) |
| Coronary heart disease | 470(28.8) | 64(88.9) |
| Liver disease | 261(16.0) | 7(9.7) |
| Renal disease | 456(27.9) | 11(15.3) |
| Malignancy | 484(29.6) | 2(2.8) |
| **Charlson index** | 6(4,8.5) | 5(4,7) |
| **Laboratory tests** |  |  |
| WBC(×10^9^/L) | 9.6(6.5,13.6) | 11.0(8.2,16.3) |
| **Neutrophil (×10^9^/L)** | 8.0(4.8,12.3) | 8.5(5.8,13.9) |
| Lymphocyte (×10^9^/L) | 1.1(0.6,1.6) | 1.0(0.5,1.6) |
| Platelet (×10^9^/L) | 206.0(133.5,286.0) | 216.0(162.0,311.0) |
| RBC (×10^12^/L) | 3.5(3.0,4.0) | 3.7(3.1,4.3) |

RBC, red blood cell; WBC, white blood cell

Table S2. Comparisons of baseline characteristics between survivors and non-survivors in each database.

|  | Mimic database | | |  | eICU database | | |
| --- | --- | --- | --- | --- | --- | --- | --- |
| Variable | Survivors | Non-survivors | *P* value |  | Survivors | Non-survivors | *P* value |
| **No. of patients, n (%)** | 1373(84.1) | 260(15.9) |  |  | 61(84.7) | 11(15.3) |  |
| **Age (years)** | 68(56,78) | 70(60,80) | 0.005 |  | 69(57,74) | 73(60,87) | 0.411 |
| **Male, n (%)** | 653(47.6) | 141(54.2) | 0.048 |  | 28(45.9) | 5(45.5) | 0.978 |
| **Infection site, n (%)** |  |  | <0.0001 |  |  |  | 0.459 |
| Respiratory infection | 279(20.3) | 97(37.3) |  |  | 23(37.7) | 7(63.6) |  |
| Urinary infection | 738(53.8) | 97(37.3) |  |  | 27(44.3) | 4(36.4) |  |
| Blood infection | 143(10.4) | 41(15.8) |  |  | 5(8.2) | 0 |  |
| Peritoneal/Pleural/Abscess/Bile infection | 83(6.0) | 11(4.2) |  |  | 2(3.3) | 0 |  |
| Other | 130(9.5) | 14(5.4) |  |  | 4(6.6) | 0 |  |
| **Co-morbidities, n (%)** |  |  |  |  |  |  |  |
| Hypertension | 532(38.7) | 84(32.3) | 0.049 |  | 15(24.6) | 2(18.2) | 0.645 |
| Diabetes | 520(37.9) | 98(37.7) | 0.956 |  | 17(27.9) | 5(45.5) | 0.293 |
| Chronic pulmonary disease | 339(24.7) | 64(24.6) | 0.979 |  | 15(24.6) | 0 | 0.105 |
| Coronary heart disease | 391(28.5) | 79(30.4) | 0.533 |  | 53(86.9) | 11(100) | 0.344 |
| Liver disease | 191(13.9) | 70(26.9) | <0.0001 |  | 6(9.8) | 1(9.1) | 1.000 |
| Renal disease | 369(26.9) | 87(33.5) | 0.030 |  | 9(14.8) | 2(18.2) | 0.672 |
| Malignancy | 403(29.4) | 81(31.2) | 0.560 |  | 2(3.3) | 0 | 1.000 |
| **Charlson index** | 6(4,8) | 7(6,9) | <0.0001 |  | 5(3.5,7) | 5(4,8) | 0.569 |
| **Laboratory tests** |  |  |  |  |  |  |  |
| WBC(×10^9^/L) | 9.3(6.5,13.4) | 10.5(6.9,14.6) | 0.014 |  | 11.1(7.8,16.8) | 10.4(8.7,13.8) | 0.820 |
| **Neutrophil (×10^9^/L)** | 7.7(4.8,11.9) | 9.6(5.1,14.7) | <0.0001 |  | 8.6(5.7,14.2) | 8.0(7.0,12.6) | 0.820 |
| Lymphocyte (×10^9^/L) | 1.1(0.6,1.6) | 0.9(0.5,1.5) | <0.0001 |  | 1.1(0.5,1.5) | 0.8(0.5,1.8) | 0.701 |
| Platelet (×10^9^/L) | 210.0(139.0,288.5) | 182.5(114.0,270.5) | 0.004 |  | 242.0(167.5,317.5) | 168.5(125.8,244.3) | 0.128 |
| RBC (×10^12^/L) | 3.5(3.0,4.0) | 3.3(2.8,3.8) | <0.0001 |  | 3.7(2.9,4.3) | 3.8(3.4,4.1) | 0.368 |
| Hemoglobin (g/dl) | 10.3(8.9,11.8) | 9.8(8.4,11.3) | <0.0001 |  | 10.3(8.6,12.0) | 11.4(10.5,11.4) | 0.074 |

RBC, red blood cell; WBC, white blood cell

Table S3. Univariate logistic regression analysis using neutrophil for hospital mortality in each database.

|  | Mimic database | | |  | eICU database | | |
| --- | --- | --- | --- | --- | --- | --- | --- |
| Variables (different level of neutrophils, ×10^9^/L) | Crude odds ratio | 95% CI | P value |  | Crude odds ratio | 95% CI | P value |
| Level 1 (0-4.8) | 1.61 | 1.05-2.47 | 0.028 |  | 1.13 | 0.16-7.98 | 0.900 |
| Level 2 (4.8-8.0) | Ref. |  |  |  |  |  |  |
| Level 3 (8.0-12.4) | 1.86 | 1.22-2.82 | 0.004 |  | 1.31 | 0.23-7.57 | 0.765 |
| Level 4 (≥12.4) | 2.65 | 1.77-3.95 | <0.0001 |  | 1.48 | 0.32-6.77 | 0.615 |

Table S4. Adjusted odds ratio using neutrophil as the design variable in multivariate logistic regression of Mimic database.

| Variable | Odds ratio | 95% CI | P value |
| --- | --- | --- | --- |
| Level 3 (8.0-12.3×10^9^/L) | 1.55 | 1.09-2.20 | 0.014 |
| Level 4 (≥12.3×10^9^/L) | 2.13 | 1.52-2.99 | <0.0001 |
| Respiratory infection | 2.75 | 2.00-3.77 | <0.0001 |
| Blood infection | 2.08 | 1.37-3.14 | 0.001 |
| RBC | 0.71 | 0.59-0.87 | 0.001 |
| Liver disease | 1.72 | 1.22-2.43 | 0.002 |
| Charlson comorbidity index | 1.15 | 1.10-1.21 | <0.0001 |

RBC, red blood cell

**Fig. S1**


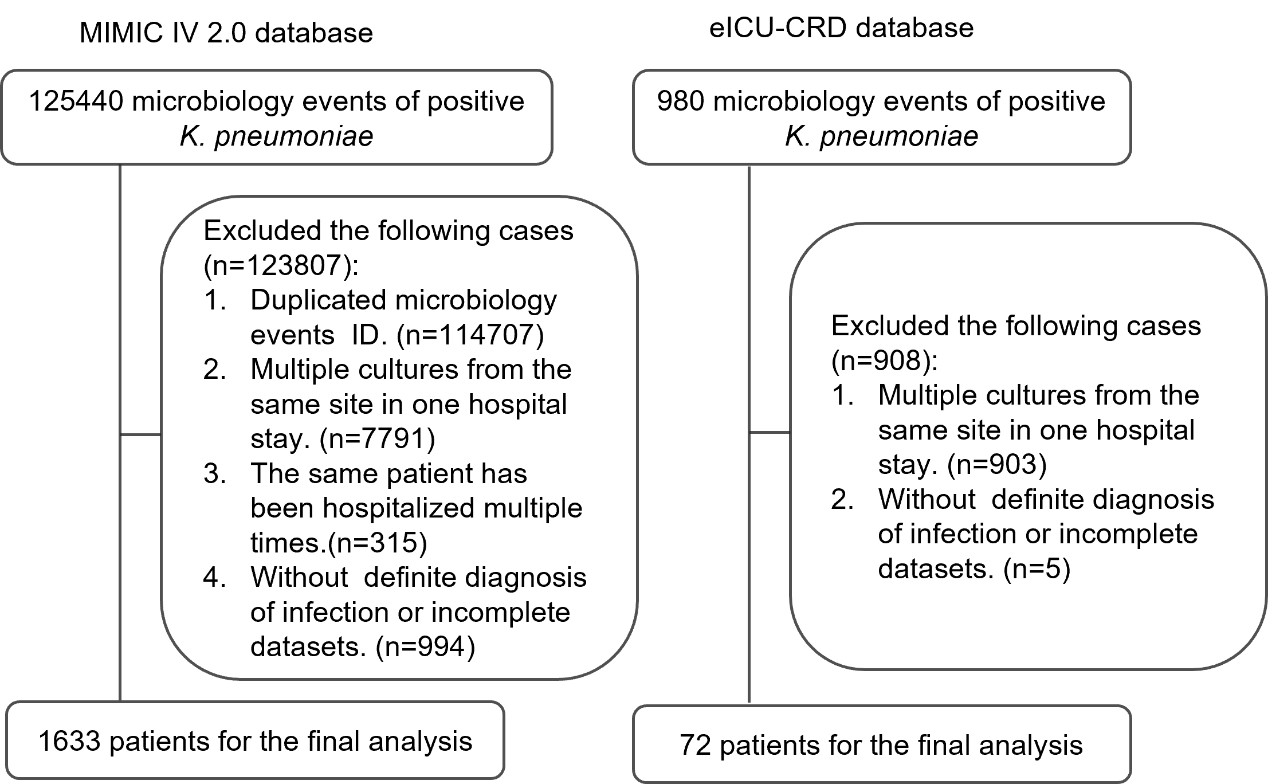


**Fig. S2**


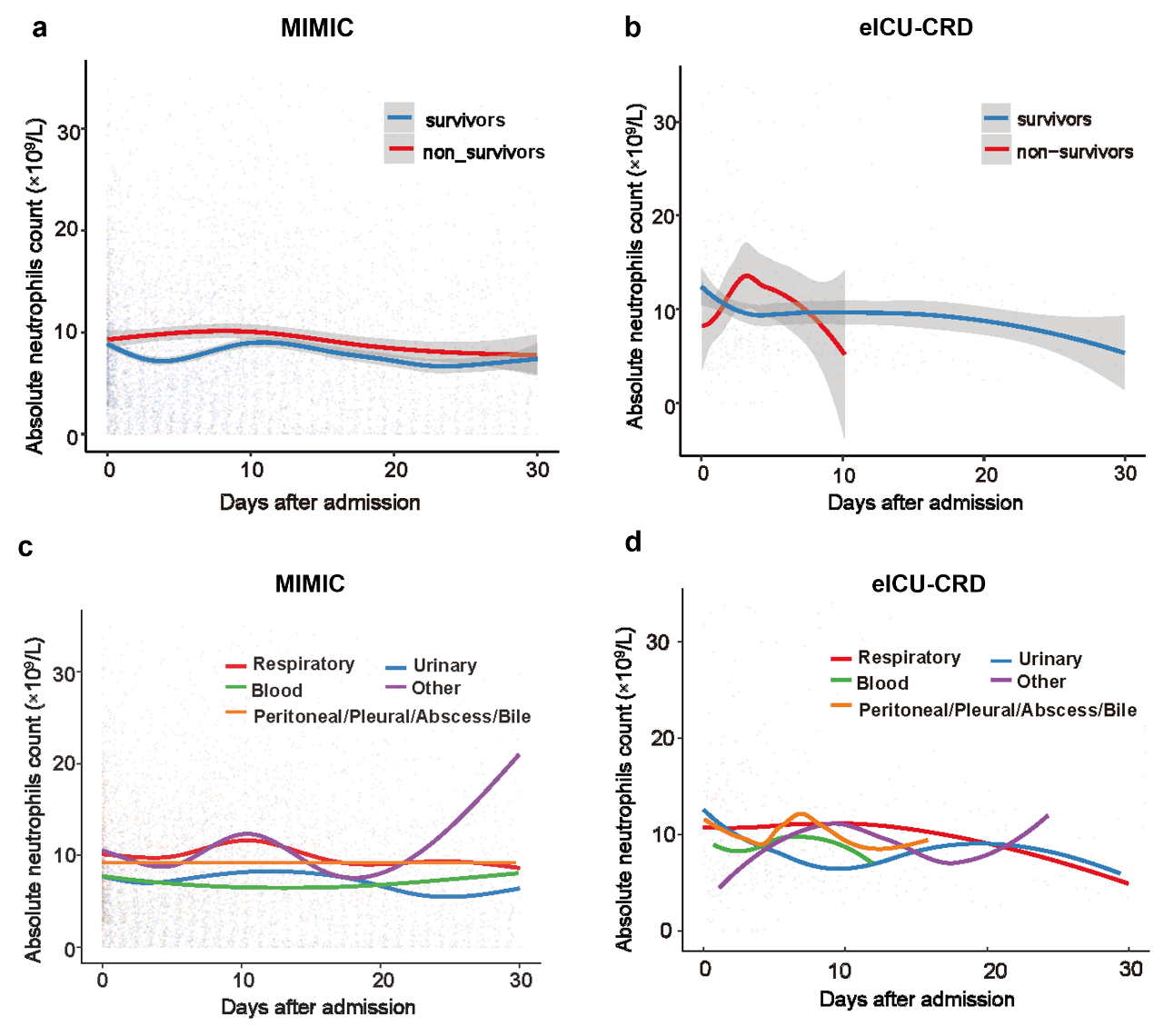


**Fig. S3**


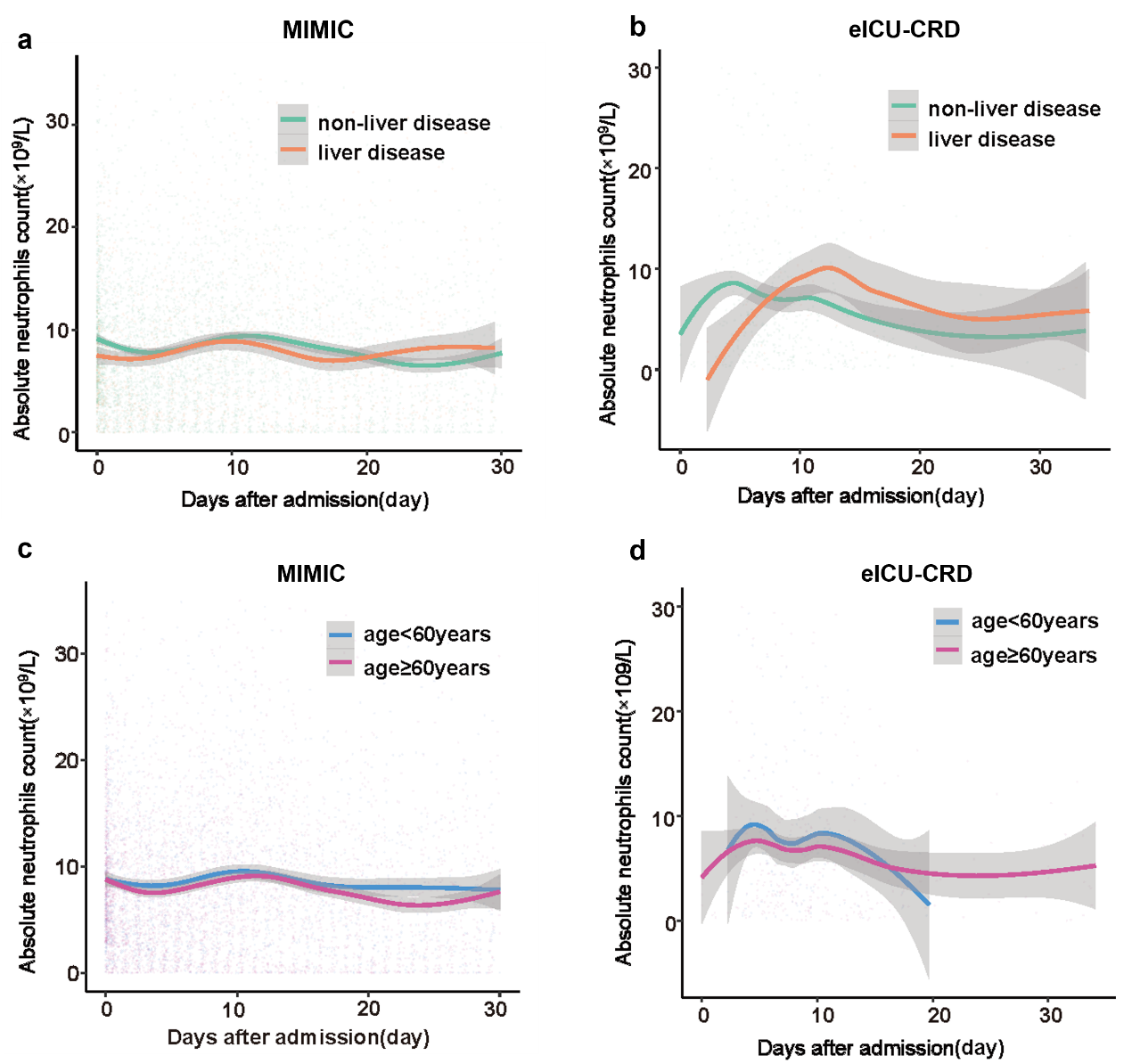


**Fig. S4**

**
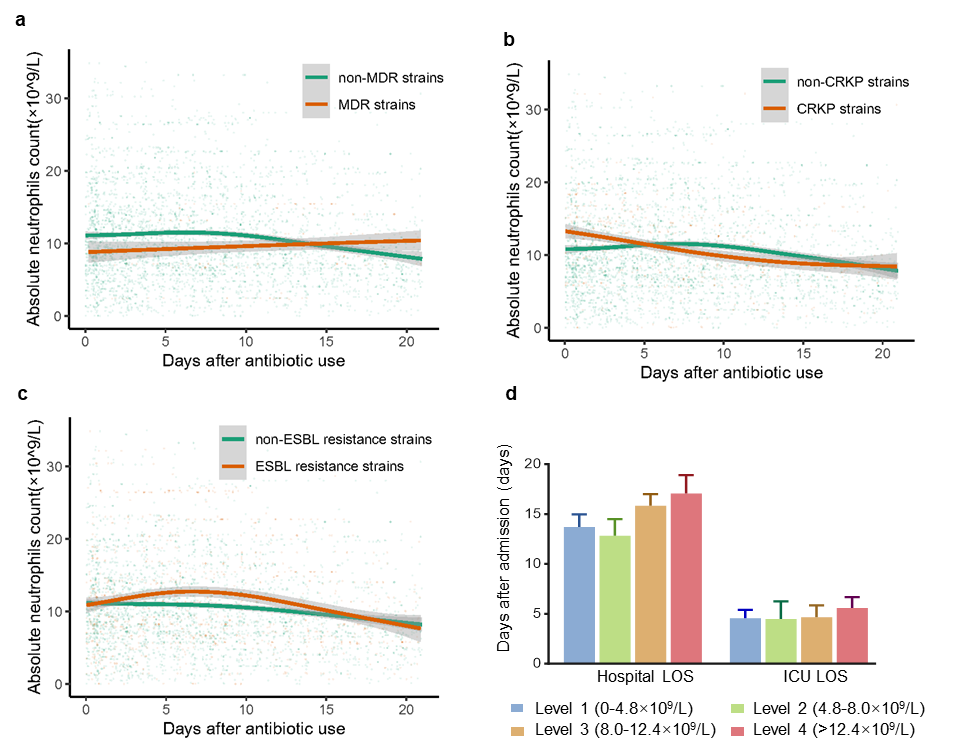
**
